# Beads-free protein immunoprecipitation for a mass spectrometry-based interactome and posttranslational modifications analysis

**DOI:** 10.1186/s12953-015-0079-0

**Published:** 2015-09-02

**Authors:** Michal Mikula, Tymon Rubel, Jakub Karczmarski, Malgorzata Statkiewicz, Karol Bomsztyk, Jerzy Ostrowski

**Affiliations:** Department of Genetics, Maria Sklodowska-Curie Memorial Cancer Center and Institute of Oncology, 02-781 Warsaw, Poland; Institute of Radioelectronics, Warsaw University of Technology, 00-665 Warsaw, Poland; Department of Medicine, University of Washington, Seattle, 98109 WA USA; Department of Gastroenterology, Hepatology and Clinical Oncology, Medical Center for Postgraduate Education, 01-813 Warsaw, Poland

**Keywords:** hnRNP K, Immuoprecipitation, Interactome, Post-translational modifications, Quantitative proteomics

## Abstract

**Background:**

Protein immunoprecipitation (IP) coupled with MS provides means to interrogate protein complexes and their posttranslational modifications (PTMs). In a typical protein IP assay antibodies are conjugated to protein A/G beads requiring large amounts of antibodies, tube transfers and centrifugations.

**Results:**

As an alternative, we present Matrix-IP, beads-free microplate-based platform with surface-immobilized antibodies. Assay utilizes standard 96-well polypropylene PCR plates that are laboratory-fabricated with UV-C light and then protein A/G coated prior to IP reaction. We demonstrate application of Matrix-IP platform in MS analysis of heterogeneous nuclear ribonucleoprotein K (hnRNP K) interactome and PTMs.

**Conclusion:**

Matrix-IP is time-saving, easy to use high throughput method adaptable for low sample amounts and automation.

**Electronic supplementary material:**

The online version of this article (doi:10.1186/s12953-015-0079-0) contains supplementary material, which is available to authorized users.

## Background

Large-scale interrogation of biological systems using mass spectrometry (MS)-based proteomics provide insights into proteins abundance, post-translational modifications (PTMs) and protein–protein interactions (PPIs). While recent advancements in the proteomic field allow to screen expression of thousands of proteins in a single shotgun MS run [1] detailed MS analysis of PTMs and PPIs is most efficiently done on protein samples purified using immunoprecipitation (IP). Protein IP with specific antibody conjugated to either protein A/G agarose or magnetic beads is the most widely used method for endogenous proteins purification and downstream analyses [2]. While relatively well established, the method requires beads-based IP, and for agarose beads involves tube transfers and centrifugations. As an alternative the beads-free microplate-based platform with surface-immobilized antibodies, called Matrix, have been used before for chromatin IP (ChIP) [3], methylated DNA IP (MeDIP) [4] and RNA IP (RIP) [5] assays (see methods section).

**Fig. 1:**
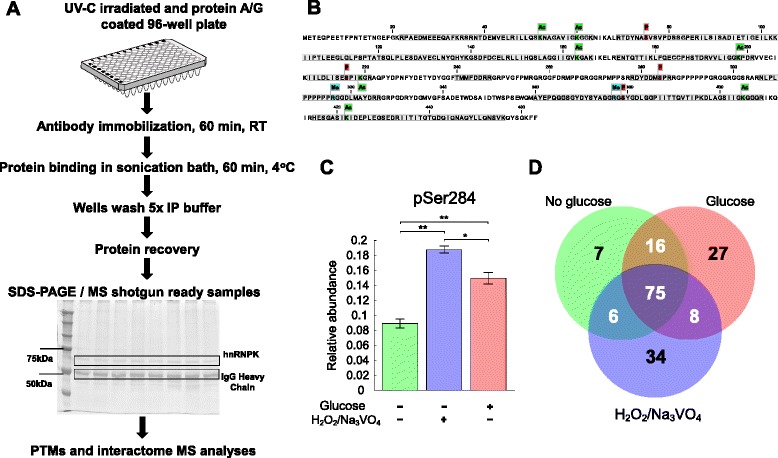
Schematic view of the microplate-based Matrix-IP method and hnRNP K peptide coverage, differential PTMs and number of proteins in the in Hepa1.6 cell interactome culture. (**a**) 96-well polypropylene microplates are irradiated with UV-C light for 48 h and coated with protein A/G, sealed, and kept at 4 °C prior to use. Detailed protocol is described the Methods section. (**b**) hnRNP K peptide coverage and localization of PTMs. Grey blocks indicate sequence covered with peptides found in MS/MS runs. Visualization of the coverage and detected modification sites were performed using CLC Sequence Viewer (CLC Bio). (**c**) K protein serine p284 phosphorylation (pSer284) changes in high glucose and oxidative stress conditions. Cells were seeded in 10 % FBS DMEM containing 4.5 g/L glucose. Next day medium was switched for 0.5 % FBS either with 4.5 g/L glucose or without glucose, and 24 h later cells without glucose were subjected to a challenge with 3.5 m M H_2_O_2_/0.1 mM Na_3_VO_4_ for 30 min. Cells were collected for protein extraction followed by hnRNP K IP reaction on Matrix-IP, SDS-PAGE and MS quantitative analyses. Bars represent mean values from five biological replicates and whiskers are for standard error of mean values. *P*-values < 0.05 were considered significant (*), (**) *p*-value < 0.0001. (**d**) Venn diagram presenting number of K protein interacting proteins in culture without, with glucose and under oxidative stress conditions. Cells were prepared as in *C* and IP reaction was performed with either unspecific IgG or anti-hnRNP K antibody followed by MS shotgun protein identification. K protein interactome, 178 proteins listed in Additional file 7: Table S6, was generated by subtracting IgG associated proteins from K protein specific bound proteins

Heterogeneous nuclear ribonucleoprotein K, hnRNP K, modular structure consists of three RNA/DNA-binding KH domains, nuclear localization signal, nuclear shuttling and K protein interactive domains [6]. K protein biological function is mostly regulated by multiple PTMs including phosphorylation [7–9], ubiquitination [10], sumoylation [11, 12] and arginine methylation [13, 14] which modulate its activity and interactions with its molecular partners. Here, we demonstrate application of beads-free microplate Matrix-IP platform combined with MS analysis to define hnRNP K PPIs and PTMs (Fig. 1a).

## Results and discussion

To define antibody binding capacity of protein A pre-coated and not coated wells we incubated (1 h) UV-treated plates with increasing amount of anti-hnRNPK antibody (epitope #54), ranging 0.5–5 μg. After washes proteins eluted from the well walls were resolved on SDS-PAGE, silver-stained and intensities of protein bands were analyzed densitometrically (Additional file 1: Figure S1A). Without protein A coating there was no IgG binding to UV-treated wells. 1 μg of #54 was a saturating amount of this antibody bound to 100 μl volume of protein A pre-coated wells. The saturating antibody amounts may differ for different antibodies and needs to be determined in each case. Further, given the fact that antibodies can be costly the tradeoff between the amount of antibody used and epitope recovery could also be a factor during optimization steps.

To assess IP efficiency of a well-bound antibody we measured depletion of hnRNP K from cell lysates after IPs. Three consecutive IP reactions carried out in triplicates were assessed by measuring input, IP and IP flow through by Western blot analysis (Additional file 1: Figure S1B). Optical density of bands showed decreasing K protein amount in flow through with consecutive IPs. After third IP reaction there was nearly 80 % K protein depletion from the starting material (Additional file 1: Figure S1B).

To assess well to well protein recovery we measured intensities of eluted proteins by MS peptide analysis. The median value of the coefficient of variation (CV) for peptide signal intensities measured in three IPed samples reached 19.06 % and 16.15 % for all and K protein peptides, respectively. After rolling up peptide-level information to proteins using the RRollup procedure [15] the median CV was equal to 17.79 %. These values indicate good reproducibility of protein recovery from wells. IP reproducibility was confirmed by the pairwise scatter plots of the measured signal intensities (Additional file 1: Figure S1C).

We illustrate application of Matrix IP by the following example. Murine Hepa 1.6 cell culture was challenged with either glucose withdrawal or oxidative stress. Matrix-IP generated samples were resolved on SDS-PAGE. HnRNP K was recovered in sufficient amounts to be visible by silver staining (Fig. 1a). K protein bands were then cut out, trypsin digested and subjected to qualitative LC-MS/MS and quantitative label-free LC-MS analyses as described before [16, 17]. For interactome studies samples recovered from IPs with either IgG control or K protein specific antibody were directly analyzed by shotgun MS.

LC-MS/MS-based analysis of 8 K protein bands yielded 46,094 fragmentation spectra and a search against the SwissProt database using the Mascot engine identified a set of 1081 peptides, with an estimated false discovery rate (FDR) of 0.01 (Additional file 2: Table S1). Among the 110 hnRNP K-derived peptides, detected in K protein bands, 13 of them contained PTM including 4, 7 and 2 phosphorylations, acetylations and methylations, respectively (Fig. 1b). Using a significance threshold of *p* ≤ 0.05, phosphorylation of serine 284, pS284, was found to be downregulated in cells growing in medium without glucose as compared to 4.5 g/L glucose, while under oxidative stress these cells exhibited significantly increased pS284 levels modification compared to cells growing in medium without or with glucose (Fig. 1c). pS284 is the most frequently reported hnRNP K’s PTM and is known to regulate multiple functions [18]. This modification is catalyzed by ERK1 and pS284 increase is associated with the nuclear export of hnRNP K and translation inhibition following a serum treatment [9]. The above experiment shows that combining Matrix IP with MS identified a key PTM.

**Fig. 2:**
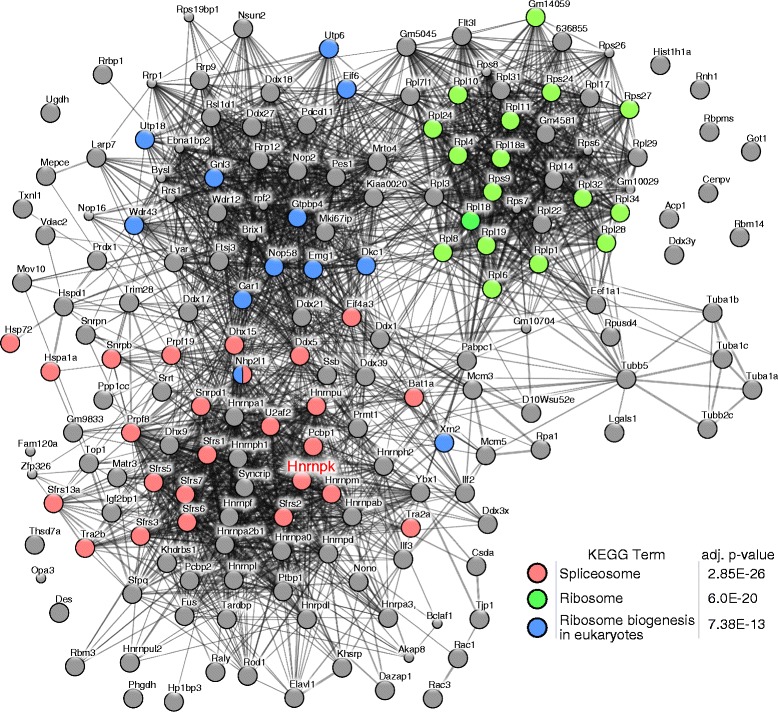
hnRNP K’s interactome and KEGG analyses with STRING database. Stronger evidences of associations are represented by thicker network edges. Node color depicts significantly enriched KEGG category assigned to a protein by STRING database [19]

Next we explored Matrix IP utility in mapping K protein interactome dynamics in the above experiment. LC-MS/MS runs of 12 mock IgG and 12 anti-hnRNP K IPed samples resulted in the acquisition of 137,162 fragmentation spectra and identification of 626 (Additional file 3: Table S2) and 1855 peptides (Additional file 4: Table S3) with FDR ≤ 0.01, respectively. 145 and (Additional file 5: Table S4) 307 (Additional file 6: Table S5) proteins were identified for IgG and K protein specific antibody, respectively. Proteins bound to IgG were then subtracted from K protein IP samples yielding a set of 178 proteins (Additional file 7: Table S6) that were represented by at least two peptides and further referenced as K protein’s interactome. Interestingly, some of these proteins were components of interactome only under specific conditions. For example there were 7, 27 and 34 proteins unique for glucose deprived, glucose and stress conditions, respectively (Fig. 1d). The molecular context of K protein interactome was next explored using the STRING database [19]. This analysis showed that hnRNP K partners are enriched for KEGG terms associated with spliceosome, ribosome and ribosome biogenesis (Fig. 2). These results are in agreement with our previous K protein interactome studies in rat hepatocyte HTC-IR [20] and human colon carcinoma HCT-116 cell line [5]. This example demonstrates that Matrix IP combined with MS and web resources can be used effectively to study protein-protein interactions.

Antibody-coated polystyrene micro-plates have been widely used in ELISA assays [21] and less frequently in standard co-IPs utilizing streptavidin or protein A/G coating [22]. Compared to polystyrene plates polypropylene plates have lower background and are more heat resistance allowing them to be used in thermal cyclers. Matrix platform, allows to carry out IPs in a compact format of 96-well PCR polypropylene plates. UV-C irradiation of PCR polypropylene plates, which normally are chemically resistant and lack protein binding capability, allows absorption of A/G protein. Polypropylene is susceptible to oxidation and UV-C light photodegradation where free radicals participate in homolytic cleavage of the carbon-hydrogen or carbon-carbon covalent bond in the polypropylene chain forming surface hydroxyl and other chemical groups [23]. These groups likely allow attachment of protein A/G molecules through intermolecular van der Waals attraction forces similarly to polystyrene microwell plates [24]. Although such a mechanism is plausible, further studies are needed to define the chemistry of protein A/G absorption to UV-treated polypropylene surface.

In summary Matrix IP using polypropylene plates has several useful features. i) Multiple IP reactions can easily be done at the same time. ii) Enough protein is captured to be detectable by SDS-PAGE and silver staining allowing MS analysis including detection of PTMs. iii) Matrix IP procedure including antibody binding to the plate, antigen IP and recovery followed by SDS-PAGE and silver staining can be done in less than 5 h. iv) UV-C polypropylene plates can be easily fabricated in the laboratory saving costs. v) 96-well PCR plate format is compatible with the thermocyclers allowing protein denaturation prior to SDS-PAGE, convenient protein alkylation, denaturation and trypsin digestion prior MS shotgun procedure. vi) Potential for high throughput automation where protein IP can be easily integrated with MS analysis using robotic devices. vii) Proteomics studies can be done in parallel and on the same plate, with ChIP and RIP analysis providing a powerful tool to facilitate integration of data from proteomic, transcriptome and epigenome studies in the same biological sample.

## Conclusions

We demonstrate application of beads-free proteins IP method using polypropylene plates for MS analyses. The advantages of plate-based antibody affinity capture include no tube transfers, low cost, speed, high-throughput, capacity for automation and ability to use it in parallel with RIP and ChIP analysis.

## Methods

### Hepa 1.6 cell line culture

Mouse Hepa 1.6 hepatocellular carcinoma cells were purchased from American Type Culture Collection (ATCC) and grown in DMEM media containing 4.5 g/L glucose (Gibco; 11965–092) supplemented with 10 % (v/v) heat-inactivated fetal bovine serum (FBS) in a humidified 5 % CO_2_ atm at 37 °C. Cells were seeded on 10 cm plates in 10 % FBS DMEM containing 4.5 g/L glucose. Next day medium was switched for 0.5 % (v/v) FBS DMEM either with glucose 4.5 g/L or glucose deprived (Gibco; 11966–025). 24 h later glucose deprived cells were subjected to an oxidative stress (3.5 mM H_2_O_2_/0.1 mM Na_3_VO_4_ for 30 min), cells were harvested and proteins were extracted.

### Protein extraction

Hepa1.6 cells (1x10^7^) were immersed in 1 mL of cold immunoprecipitation (IP) buffer [150 mM NaCl, 5 mM EDTA, 1 % Triton X-100, 0.5 % NP-40, 50 mM Tris–HCl (pH 7.5)] containing cocktail of protease and phosphatase inhibitors (Thermo; 78441) and then kept on ice for 5 min. Next, extracts were subjected to homogenization at 4 °C in a Bioruptor Plus (Diagenode) using a 30 s on-off cycle for 10 min at low intensity followed by centrifugation at 12 000 × g for 15 min in 4 °C. 200 μL of supernatant was saved to measure total protein concentration followed by SDS-PAGE. Remaining 800 μL of the extract was immediately used in IP reaction.

### hnRNP K IP

hnRNP K protein IP was performed using the Matrix-ChIP platform as previously described [3] with modifications. Briefly, 96-well polypropylene microplate (MicroAmp® Optical 96-Well Reaction Plate-Life Technologies) was irradiated with UV-C light (Three Philips TUV TL-D 15 W UV-C compact bulbs connected to Philips HF-P 3/418 TLD E II stabilizer) for 48 h. Plates were then coated with protein A (Sigma; P7837) by incubating overnight at room temperature with 200 μL of 2 μg/mL protein A in PBS per well and then stored for at least a day at 4 °C prior to use (Fig. 1a). Plates sealed with adhesive plastic sheets were stored at 4 °C for several weeks. On the day of use, plates were washed with 200 μL PBS per well and wells were incubated with 0.5 μg of anti-hnRNPK (epitope #54) or unspecific rabbit polyclonal IgG (I-1000; Vector labs) antibody in 100 μL IP buffer for 60 min at room temperature. Antibody was aspirated and whole cell culture extracts were aliquoted on ice into eight wells in a vertical row as 100 μL portions. 12 and 24 samples were simultaneously processed on 96-well plate for SDS-PAGE and MS shotgun experiment, respectively. Plate was covered with an adhesive plastic film and IP of antigen to well walls was performed by floating the plate in ultrasonic water bath (Branson 3510) for 60 min at 4 °C. Wells were washed five times with ice-cold IP buffer. To elute proteins from wells 50 μL of either 1 × Laemmli sample buffer (for SDS-PAGE) or 0.1 % TFA (for MS shotgun) was applied to one of the border wells and all wells were sealed with flat 8-cap strip. Plate was mixed, MixMate (Eppendorf) for 30 s at 1700 rpm, and extracted sample was transferred to adjacent well using a multichannel pipette. The extraction was repeated with same solution for all remaining wells in a vertical row where IP reactions occurred for a given sample. Samples for MS shotgun survey were neutralized by adding 1 M NH_4_HCO_3_ solution to a final 0.1 M concentration and then were reduced, alkylated, and trypsin-digested using standard protocols. Samples for SDS-PAGE were heated at 99 °C in thermocycler for 5 min and separated by 10 % SDS-PAGE and silver stained. Gel fragments containing K protein bands were cut out separately for each electrophoresis line; proteins in the gel were reduced, alkylated, and trypsin-digested using standard protocols, and the resulting peptides were extracted using 0.1 % TFA/2 % acetonitrile (ACN).

### LC-MS settings

LC-MS analysis of peptides was performed on a LTQ-Orbitrap Elite mass spectrometer (Thermo Scientific) coupled with a nanoAcquity (Waters Corporation) LC system. Spectrometer parameters were as follows: polarity mode, positive; capillary voltage, 2 kV. A sample was first applied to the nanoACQUITY UPLC Trapping Column (Waters) using water containing 0.1 % formic acid as the mobile phase. Next, the peptide mixture was transferred to the nanoACQUITY UPLC BEH C18 Column (Waters, 75 μm inner diameter; 250 mm long) and an ACN gradient (5–30 % over 45 min) was applied in the presence of 0.1 % formic acid with a flow rate of 250 nL/min and eluted directly to the ion source of the mass spectrometer. Each LC run was preceded by a blank run to avoid sample carry-over between the analyses.

Qualitative LC-MS/MS analyses were performed on pooled samples in data-dependent acquisition mode and high-energy collision dissociation (HCD) was used for peptide fragmentation. Quantitative analyses of individual samples were performed by using separate survey scan LC-MS runs with resolving power set to 30 000, m/z measurement range of 300–2 000 and the same ACN gradient settings as those used for the LC-MS/MS runs.

### Qualitative MS data processing and database search

The acquired MS/MS raw data files were preprocessed with Mascot Distiller (version 2.5.1, Matrix Science), and the resulting peak lists were submitted to the Mascot engine (version 2.4.1, Matrix Science) and searched against the SwissProt *Mus musculus* database (release 2014.11) concatenated with SwissProt *Oryctolagus cuniculus* IgG entries, and contaminant proteins retrieved from the common Repository of Adventitious Proteins (cRAP, http://www.thegpm.org/crap/index.html). In total, the database included 16,836 target sequences, and the same number of reversed decoy records. The search parameters were as follows: enzyme specificity: semitrypsin; maximum number of missed cleavages: 1; protein mass: unrestricted; parent ions mass error tolerance: 5 ppm; fragment ions mass error tolerance: 0.02 Da; fixed modifications: Carbamidomethylation (C); variable modifications: Acetyl (K) (42.010565 Da), Methyl (K) (14.015650 Da), Methyl (R) (14.015650 Da), Dimethyl (R) (28.031300 Da), Trimethyl (R) (42.046950 Da), Deamidated (R) (0.984016 Da), Phospho (ST) (79.966331 Da), Phospho (Y) (79.966331 Da) and Oxidation (M) (15.994915 Da). The statistical significance of identified peptides was determined using a target/decoy database search approach and a previously described procedure that provided *q*-value estimates for each peptide spectrum match (PSM) in the data set [25, 26]. Only PSMs with *q*-values ≤ 0.01 were regarded as confidently identified. Furthermore, all the peptide sequences matched to database entries representing contaminant proteins were rejected.

Additional acceptance criteria were used for assessing confidence of modified peptides. In the first step, the exact position of the modifications in the sequence was established by an adopted version of the phosphoRS algorithm [27]. Next, selected types of sites were rejected as potential experimental artifacts. Those included: lysine methylations on the C-terminus of the sequence or detected in peptides with acidic residues (possible artifacts of methyl esterification of the carboxylic group) and peptides with deamidation on the C-terminal arginine (tryptic cleavage after a deamidated residue have been recently shown as a highly unlikely event [28]).

Proteins represented by less than two peptides, or present in less than two LC-MS/MS runs were excluded from further analysis. Proteins identified by a subset of peptides from other proteins were filtered out from the results, and those matching the same set of peptides were grouped together into clusters. All the steps involved in Mascot results processing were performed using MScan, a proprietary Java application available at http://proteom.ibb.waw.pl/mscan. CLC Sequence Viewer (CLC Bio) was used for the visualization of the detected post-translational modification sites.

### Quantitative MS data processing

Peptides identified in all LC-MS/MS runs were merged into a common list, which was next overlaid onto 2-D maps generated from the LC-MS profile data of individual samples. The extraction procedure was described in detail in a previous study [29]. Briefly, the list of identified peptides was used to tag the corresponding peptide-related ion spectra based on m/z differences, deviations from the predicted elution times, and the match between the theoretical and observed isotopic envelopes. The maximum deviation accepted in m/z and the retention time was established separately for each of the processed LC-MS spectra to account for possible variations in mass measurement accuracy and chromatographic separation between runs. Peptide ion abundances were determined as the heights of 2-D fits to the most prominent peaks of the tagged isotopic envelopes. For the quantitative analysis of post-translational modification sites ratios of modified to unmodified peptide-pairs were calculated.

### Statistical analysis of quantitative MS measurements

Pair-wise comparisons of post-translational modification levels in Hepa 1.6 cells were carried out using the *t*-test. Modification sites with *p*-values ≤ 0.05 were considered as significantly changed.

## Additional files

Additional file 1:
**Figure S1.** Technical characterization of IP using antibody-coated polypropylene well (**A**). Determination of IgG saturating amount. The UV-activated polypropylene and protein A coated (2 μg/mL) wells were incubated for 1 h with increasing amounts of anti-hnRNP K antibody (epitope #54), ranging 0.5–5 μg (with 0.5 μg increment) in 100 μL of IP buffer. Antibody containing IP-buffer was next aspirated and wells washed three times with a 100 μL of IP buffer. Well-bound antibody was eluted with 1 × Laemmli sample buffer, resolved on SDS-PAGE and silver-stained. The intensity of protein bands was analyzed densitometrically with OptiQuant (Packard Instrument) software. Data are presented as fold increase relative to 0.5 μg amount (*n* = 2; mean ± SD). (**B**) Efficacy of hnRNP K depletion with well-bound antibody. Total protein extract from 3x10^5^ Hepa 1.6 cells was used in three consecutive IP in wells conjugated with 0.5 μg #54 antibody. Total, IP and IP flow through (Ft) samples were resolved on SDS-PAGE followed by Western blot analysis with #54 antibody (*upper panel*). The intensities of hnRNP K bands in Ft samples were analyzed densitometrically with OptiQuant (*n* = 3; mean ± SD) (*lower panel*). (**C**) Pairwise scatter plots of MS-measured peptide ion intensities in three independent IP reactions. The IP reaction was performed as in *B* with protein recovery from wells with 0.1 % TFA and then subjected to MS analysis. The values are log-transformed (base 10). Red points indicate peptides originating from the hnRNP K protein. (PDF 822 kb) Additional file 2:
**Table S1.** List of peptides and their characteristic identified in K protein bands from SDS-PAGE. (XLSX 69 kb) Additional file 3:
**Table S2.** List of peptides and their characteristic identified in plain IgG antibody immunoprecipitates. (XLSX 44 kb) Additional file 4:
**Table S3.** List of peptides and their characteristic identified in K protein specific antibody (epitope #54) immunoprecipitates. (XLSX 113 kb) Additional file 5:
**Table S4.** The list of proteins in the IgG immunoprecipitated samples identified with at least 2 peptides and information on identified proteins including number of peptides identified per protein and the percent of coverage. Proteins matching to the same sets of peptides were grouped. (XLSX 16 kb) Additional file 6:
**Table S5.** The list of proteins in the anti-hnRNP K (epitope #54) immunoprecipitated samples identified with at least 2 peptides and information on identified proteins including number of peptides identified per protein and the percent of coverage. Proteins matching to the same sets of peptides were grouped. (XLSX 26 kb) Additional file 7:
**Table S6.** HnRNP K’s interactome in Hepa 1.6 cells under three different conditions. Proteins matching to the same sets of peptides were grouped. Numbers are the peptides matching a given protein under a specific condition. (XLSX 16 kb)

## Abbreviations

hnRNP K: Heterogeneous nuclear ribonucleoprotein K
LC-MS/MS: Liquid chromatography followed by tandem mass spectrometry
PTM: Post-translational modification
IP: Immunoprecipitation
PPIs: Protein–protein interactions
ChIP: Chromatin immunoprecipitation
MeDIP: Methylated DNA immunoprecipitation
RIP: RNA immunoprecipitation
KEGG: Kyoto Encyclopedia of Genes and Genomes
