# Supplementary figures and images for: Beads-free protein immunoprecipitation for a mass spectrometry-based interactome and posttranslational modifications analysis
Source: Proteome Sci. 2015 Sep 2;13:23. doi: 10.1186/s12953-015-0079-0 (PMC4557753; doi:10.1186/s12953-015-0079-0)

**A**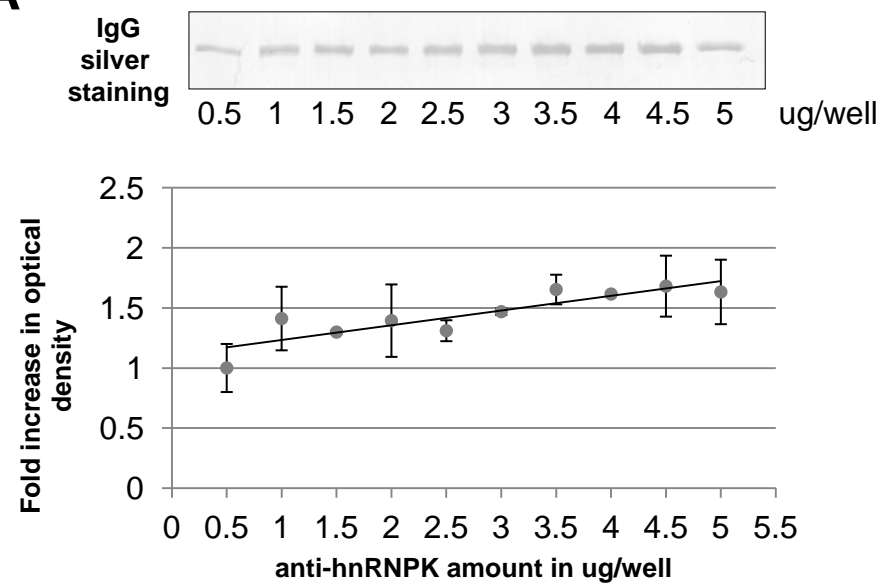**B**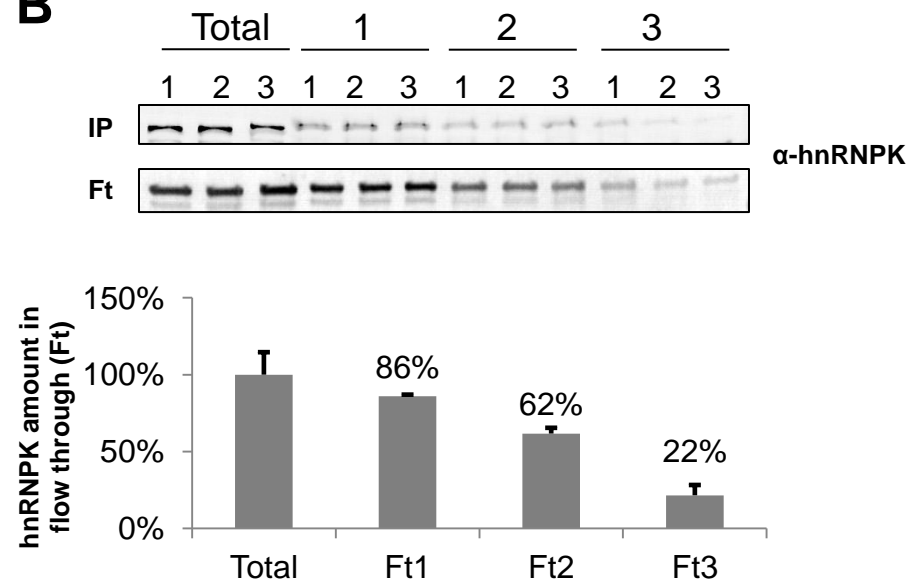**C**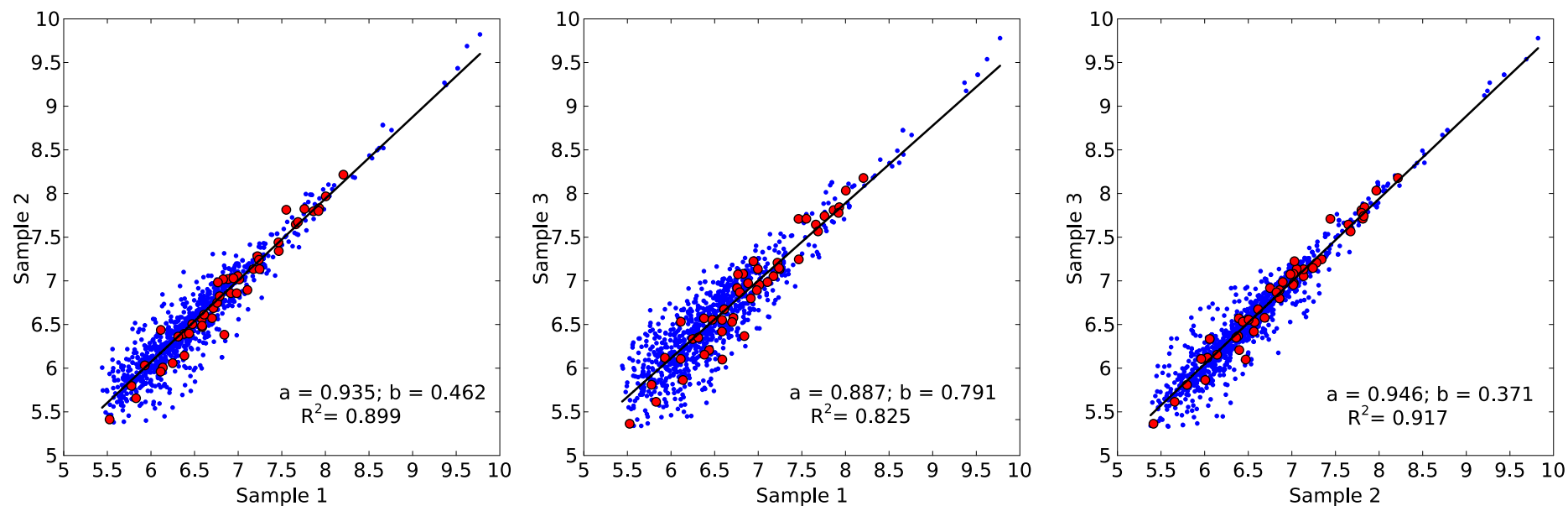

Supplement: Additional file 1: — Figure S1. Technical characterization of IP using antibody-coated polypropylene well (A). Determination of IgG saturating amount. The UV-activated polypropylene and protein A coated (2 μg/mL) wells were incubated for 1 h with increasing amounts of anti-hnRNP K antibody (epitope #54), ranging 0.5–5 μg (with 0.5 μg increment) in 100 μL of IP buffer. Antibody containing IP-buffer was next aspirated and wells washed three times with a 100 μL of IP buffer. Well-bound antibody was eluted with 1 × Laemmli sample buffer, resolved on SDS-PAGE and silver-stained. The intensity of protein bands was analyzed densitometrically with OptiQuant (Packard Instrument) software. Data are presented as fold increase relative to 0.5 μg amount (n = 2; mean ± SD). (B) Efficacy of hnRNP K depletion with well-bound antibody. Total protein extract from 3x10^5^ Hepa 1.6 cells was used in three consecutive IP in wells conjugated with 0.5 μg #54 antibody. Total, IP and IP flow through (Ft) samples were resolved on SDS-PAGE followed by Western blot analysis with #54 antibody (upper panel). The intensities of hnRNP K bands in Ft samples were analyzed densitometrically with OptiQuant (n = 3; mean ± SD) (lower panel). (C) Pairwise scatter plots of MS-measured peptide ion intensities in three independent IP reactions. The IP reaction was performed as in B with protein recovery from wells with 0.1 % TFA and then subjected to MS analysis. The values are log-transformed (base 10). Red points indicate peptides originating from the hnRNP K protein. (PDF 822 kb) [file 12953_2015_79_MOESM1_ESM.pdf]
